# The Impact of Cardiac-induced Post-traumatic Stress Disorder Symptoms on Cardiovascular Outcomes: Design and Rationale of the Prospective Observational Reactions to Acute Care and Hospitalizations (ReACH) Study

**DOI:** 10.5334/hpb.16

**Published:** 2019-01-14

**Authors:** Jeffrey Birk, Ian Kronish, Bernard Chang, Talea Cornelius, Marwah Abdalla, Joseph Schwartz, Joan Duer-Hefele, Alexandra Sullivan, Donald Edmondson

**Affiliations:** * Columbia University Medical Center, US; † Stony Brook University, US

**Keywords:** acute coronary syndrome, posttraumatic stress disorder, cardiovascular disease, emergency department, psychosocial factors, medication adherence

## Abstract

**Aims:**

As many as 1 in 8 acute coronary syndrome (ACS) patients develop posttraumatic stress disorder (PTSD) due to the ACS, and ACS-induced PTSD may increase secondary cardiovascular disease (CVD) risk. However, prior studies have been small and underpowered to test plausible behavioral or biological mechanisms of the hypothesized PTSD-secondary CVD risk association. In this paper, we describe the design and methods of a large prospective observational cohort study to estimate the prognostic significance of ACS-induced PTSD, mechanisms for its association with CVD risk, and emergency department (ED) factors that may increase PTSD risk, in a cohort of patients evaluated for acute coronary syndrome (ACS) in the ED of a large, urban academic medical center.

**Methods:**

The Reactions to Acute Care and Hospitalization (ReACH) study follows 1,741 racially, ethnically, and socioeconomically diverse patients initially presenting to the ED with ACS symptoms. Psychosocial factors are assessed at baseline. Medication adherence is monitored by electronic pill bottle (eCAP). Participants are contacted by phone at 1-, 6-, and 12-months post-hospitalization to assess PTSD symptoms, hospital readmission, and recurrent CVD events/mortality (proactively searched and confirmed by medical records).

**Conclusion:**

This study will provide the most accurate estimates to date of PTSD’s association with recurrent CVD events and mortality and will test whether medication adherence mediates that association. Further, it will provide estimates of the contribution of ED and hospital factors to PTSD risk in ACS patients. If our hypotheses are supported, we will have identified PTSD as a novel target for secondary risk reduction.

Over one million patients in the United States are hospitalized annually with acute coronary syndrome (ACS), including non-ST segment elevation myocardial infarction (NSTEMI) or unstable angina (UA) (Benjamin et al., 2017). Despite improved survival rates in recent years due to improvements in medical care (McManus et al., 2011), recurrent cardiovascular event and mortality risk remains high for NSTEMI/UA patients (Sanchis-Gomar, Perez-Quilis, Leischik, & Lucia, 2016). ACS patients are not only at risk for recurrent cardiac events and mortality. For many, the ACS is a frightening experience associated with significant pain and fear of dying (von Känel et al., 2011; Whitehead, Strike, Perkins-Porras, & Steptoe, 2005), and may induce long-lasting symptoms of post-traumatic stress disorder (PTSD) (Abbas et al., 2009), a psychiatric disorder characterized by psychological distress and impaired functioning (Cohen et al., 2009).

## Investigating the link between ACS-induced PTSD and recurrence of cardiovascular events

PTSD is diagnosed when an individual experiences four categories of symptoms after exposure to a life-threatening traumatic event: persistent re-experiencing of the event; efforts to avoid stimuli related to the event; negative alterations in cognition and mood; and alterations in arousals and reactivity (American Psychiatric Association, 2013). In 1994, the Diagnostic and Statistical Manual of Mental Disorders (DSM-IV) included life-threatening ill-nesses as a type of traumatic event that could induce PTSD (American Psychiatric Association, 1994). Since then, a growing number of researchers have evaluated the incidence of PTSD among ACS survivors (Spindler & Pedersen, 2005; Tedstone & Tarrier, 2003). A meta-analysis of 24 studies revealed that ACS-induced PTSD is common, present in 12% (95% confidence interval [CI], 9%–16%) of ACS survivors (Edmondson et al., 2012).

ACS-induced PTSD may also increase cardiovascular risk. An emerging body of literature shows that PTSD due to non-medical events is associated with increased incidence of coronary heart disease (CHD) in initially healthy adults (Edmondson & Cohen, 2013; Edmondson, Kronish, Shaffer, Falzon, & Burg, 2013; Vaccarino et al., 2013). Few studies, however, have assessed the impact of ACS-induced PTSD on subsequent prognosis. A recent meta-analysis comprised of just 3 studies and 617 total patients suggested that PTSD may double the risk of recurrent cardiovascular events/mortality (Edmondson et al., 2012). The evidence for this link requires further study, however, due to limitations in the supporting research to date. First, the number of studies in this preliminary meta-analysis is quite low. Second, one of the three studies had a non-significant increased risk for the overall measure of PTSD symptoms and instead showed an association specific to the intrusions subscale of PTSD (Edmondson et al., 2011). Third, one of the studies had a particularly small sample size (*N* = 73) (Shemesh et al., 2004).

Several mechanisms have been proposed to explain how PTSD could increase cardiovascular risk. Among the most promising are behavioral pathways. Patients with PTSD are typically burdened by intrusive memories of the traumatic event, and consciously or unconsciously attempt to avoid reminders of this experience. Thus, when PTSD is induced by an ACS, survivors might avoid taking the medications that remind them of their heart disease. In support of this hypothesis, prior studies have shown that patients with stroke-induced PTSD report increased concerns about their medications but not decreased awareness of their necessity for secondary prevention, suggesting an ambivalent attitude towards medications which could be another manifestation of an avoidance phenomenon (Edmondson, Horowitz, Goldfinger, Fei, & Kronish, 2013). We and others have begun to explore whether ACS-induced PTSD is indeed associated with lower adherence to cardioprotective medications. In a small study of 64 post-MI patients, patients with MI-induced PTSD were more likely to be non-adherent to aspirin as measured by platelet function testing (Shemesh et al., 2004). In a sample of 535 stroke survivors, we showed that those with “likely” stroke or transient ischemic attack (TIA)-induced PTSD had nearly 3 times the odds of self-reported non-adherence to medications compared to stroke survivors without PTSD (Kronish, Edmondson, Goldfinger, Fei, & Horowitz, 2012). We also showed in a cohort of primary care patients followed at Veterans Administration clinics that those with PTSD diagnosis were more likely to report missing or skipping their medications, consistent with our avoidance hypothesis (Kronish, Edmondson, Li, & Cohen, 2012). Not all studies, however, have found an independent association between PTSD and medication adherence. In a cohort of patients with stable CHD, for example, PTSD was not associated with medication adherence after adjusting for depression (Zen, Whooley, Zhao, & Cohen, 2012).

Many patients who present to the ED with acute chest pain are ultimately ruled out for ACS. A number of studies have described the psychological impact of ACS (Cohen, Edmondson, & Kronish, 2015). Much less is known about the experience of patients who present to the ED with probable ACS, but who subsequently rule out after diagnostic testing. Of all the patients who present to the ED with ACS-like symptoms (e.g., chest pain, shortness of breath, left arm pain, in the presence of documented risk factors for ACS), less than half are ultimately diagnosed with an ACS (Goodacre et al., 2005). Most patients who rule out for ACS receive non-life-threatening diagnoses for their presenting symptoms, such as musculoskeletal pain or gastroesophageal reflux disorders, while others do not receive any specific diagnosis (Chambers, Marks, & Hunter, 2015). Prior studies suggest that patients with a non-cardiac etiology for their ACS-like symptoms have increased health care utilization and costs, but they are not at increased risk of subsequent cardiovascular events (Fagring et al., 2010). Because of the relatively benign medical prognosis for patients who rule out for ACS, physicians may see little cause for concern once patients rule out. However, from the point of view of the patient, the experience of presenting to the hospital with ACS symptoms may still be traumatic, irrespective of the final diagnosis they receive. Indeed, patients often do not know whether their ACS symptoms were ultimately diagnosed as ACS (Yasaitis, Berkman, & Chandra, 2015). PTSD is now commonly understood to be induced by the perception of life threat during a traumatic event, regardless of the objective risk, particularly for potentially life threatening medical events (Laubmeier & Zakowski, 2004). Accordingly, patients who rule out for ACS may still be at risk for developing PTSD due to the event. Many of these patients have underlying CVD risk factors that led to concern for ACS in the first place, and such patients who develop PTSD symptoms may similarly be at risk for avoiding their cardiovascular medications. No study has tested whether PTSD induced by a suspected but unconfirmed ACS can influence medication adherence or CVD risk and mortality.

## Risk factors for ACS-induced PTSD

Although risk factors for PTSD due to stereotypically PTSD-inducing events such as combat and assault are well described, less is known about the factors that predict PTSD after ACS. Identifying risk factors is necessary for guiding development of interventions to prevent ACS-induced PTSD in patients at highest risk. If PTSD also increases secondary CVD risk, then interventions that prevent PTSD may have collateral benefits on reducing CVD events and mortality in ACS patients as well.

There are many factors during the initial ED visit that can induce stress and may influence the development of PTSD. These can be subsumed under three categories: environmental, personal, and social, each of which is assessed in the present study. To our knowledge, few prior studies have assessed the role of these ED factors in predicting ACS-induced PTSD. Accordingly, there is a need to comprehensively assess the role of the ED environment (i.e., crowding, wait time, exposure to critical care), reactivity of the patient (physiological or psychological), and interpersonal aspects of the patient experience (doctor-patient communication, social support) with respect to subsequent ACS-induced PTSD risk. Each set of factors has putative and/or demonstrated links with PTSD and/or adverse health events.

A number of characteristics of the ED environment have been associated with psychological and cardiovascular outcomes. ED crowding tends to increase wait times and is a source of added stress among patients already experiencing alarming levels of cardiac distress (Kellermann, 2006). ED crowding has also been associated with increased risk of poor cardiac outcomes (Pines, Hollander, Localio, & Metlay, 2006) and one-year mortality (Shen & Hsia, 2011). In addition to long-term cardiac outcomes, ED crowding also predicts serious psychological distress in the form of more severe PTSD symptoms in NSTEMI/UA patients (Edmondson, Shimbo, Ye, Wyer, & Davidson, 2013). Exposure to potentially traumatic critical care procedures of other patients in the ED has also been associated with poorer cardiovascular outcomes (Fishman et al., 2006).

Individual differences in psychological and physiological reactivity among ACS patients in the ED are also essential factors to assess when considering the development of ACS-induced PTSD. Psychological responses to ACS such as anxiety during the event predict elevated PTSD symptoms (Rocha et al., 2008). In fact, fear of dying and helplessness are better predictors of who will develop PTSD than common metrics of the severity of the cardiac event (e.g., left ventricular ejection fraction, number of coronary occlusions) (Guler et al., 2009). In non-ACS populations, physiological responses such as increased heart rate have also been associated with the development of PTSD (Zatzick et al., 2005).

Interpersonal factors constitute another set of ED factors that are putatively related to psychological and physical health outcomes. Lack of social support from family or friends in the ED predicts worse cardiovascular outcomes in the short term such as higher blood pressure and heart rate in response to stress (Thorsteinsson & James, 1999), and low social support is an established risk factor for PTSD in patients who experience myocardial infarction (Bennett, Owen, Koutsakis, & Bisson, 2002). Beyond the impact of friends and family, lower quality relationships between patients and doctors are associated with poorer health outcomes (Ong, De Haes, Hoos, & Lammes, 1995). Although research on the quality of patient-doctor communication in the ED and subsequent PTSD is limited, it is likely that poor patient-doctor communication is relevant to psychological outcomes (Chang, Sumner, Haerizadeh, Carter, & Edmondson, 2016).

Given the multiple variables and pathways that may contribute to the development of ACS-induced PTSD in the acute care environment, the efficient and systematic evaluation of these potential relationships raises numerous methodological challenges. The assessment of ACS-induced PTSD and its associated psychological and cardiovascular outcomes involves the collection of data across multiple timepoints and clinical contexts (e.g., ED, inpatient setting, at-home treatment). Difficulties may also exist for the efficient enrollment and follow-up of patients treated for life-threatening disease in the often chaotic ED setting. Additionally, it is necessary to collect time-sensitive, ecological information from patients while they are still in the ED setting being evaluated for ACS. Interviewing patients while undergoing ACS evaluation in the ED allows one to assess ED, patient, and interpersonal factors during that heightened period of uncertainty that may influence patients’ long-term psychological adjustment.

**Figure 1:**
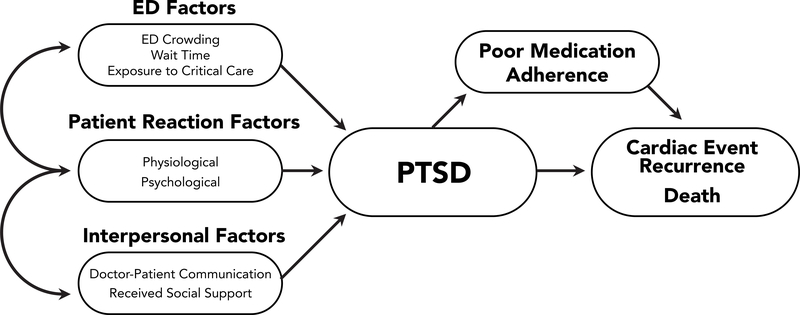
Proposed theoretical model for investigating potential pathways connecting factors at the time of the initial emergency department (ED) visit in patients presenting with symptoms of acute coronary syndrome. ED factors in three domains predict subsequent post-traumatic stress disorder (PTSD), which predicts medical outcomes that may be partially mediated by changes in medication adherence.

In this paper, we describe the design and methods of the Reactions to Acute Care and Hospitalizations (ReACH) study, a prospective observational cohort study that enrolls patients being evaluated in the ED for ACS, and follows them for 1 year to PTSD and CVD/mortality outcomes. The ReACH study tests a theoretical model in which ED factors contribute to the development of post-traumatic stress disorder (PTSD), which may in turn influence prognosis, including risk for cardiac event recurrence and mortality. We also test one potential mechanism by which PTSD symptoms may increase risk for health events: poor medication adherence (see Figure 1 for a depiction of the framework of tested associations emerging over time).

The study aims are as follows: (1) to test whether suspected ACS-induced PTSD is associated with increased risk of mortality and recurrent major adverse cardiac events (MACE) at 1 year; (2) to determine whether electronically-measured medication nonadherence mediates any association of suspected ACS-induced PTSD with CVD risk; (3) to identify predictors of suspected ACS-induced PTSD that are related to the patient, the physicians, and the ED environment. Not all patients who are initially evaluated for ACS in the ED will later be confirmed to have ACS. Therefore our study data provide an opportunity to determine whether patients who “rule out” for ACS are similarly at increased risk of PTSD and adverse CVD outcomes as patients with confirmed ACS. Thus, an exploratory aim of the study is to test whether risk for these adverse outcomes differs according to true ACS vs. ACS rule-out status. By sharing our study design, we hope to present an efficient and efficacious model for conducting prospective observational work in acute care environments such as the ED.

## Methods

Participants enrolled in this study are patients who present to the Columbia-New York Presbyterian Hospital ED with chest pain and a suspected diagnosis of NSTEMI or UA during initial presentation (see below for inclusion/exclusion criteria). Patients with STEMI were not enrolled as these patients are typically rushed to the cardiac catheterization lab before ED assessments can be completed, a key component of the study design. Columbia-New York Presbyterian Hospital is a quaternary academic medical center serving a dense urban area. The hospital has over 100,000 ED visits annually.

**Figure 2:**
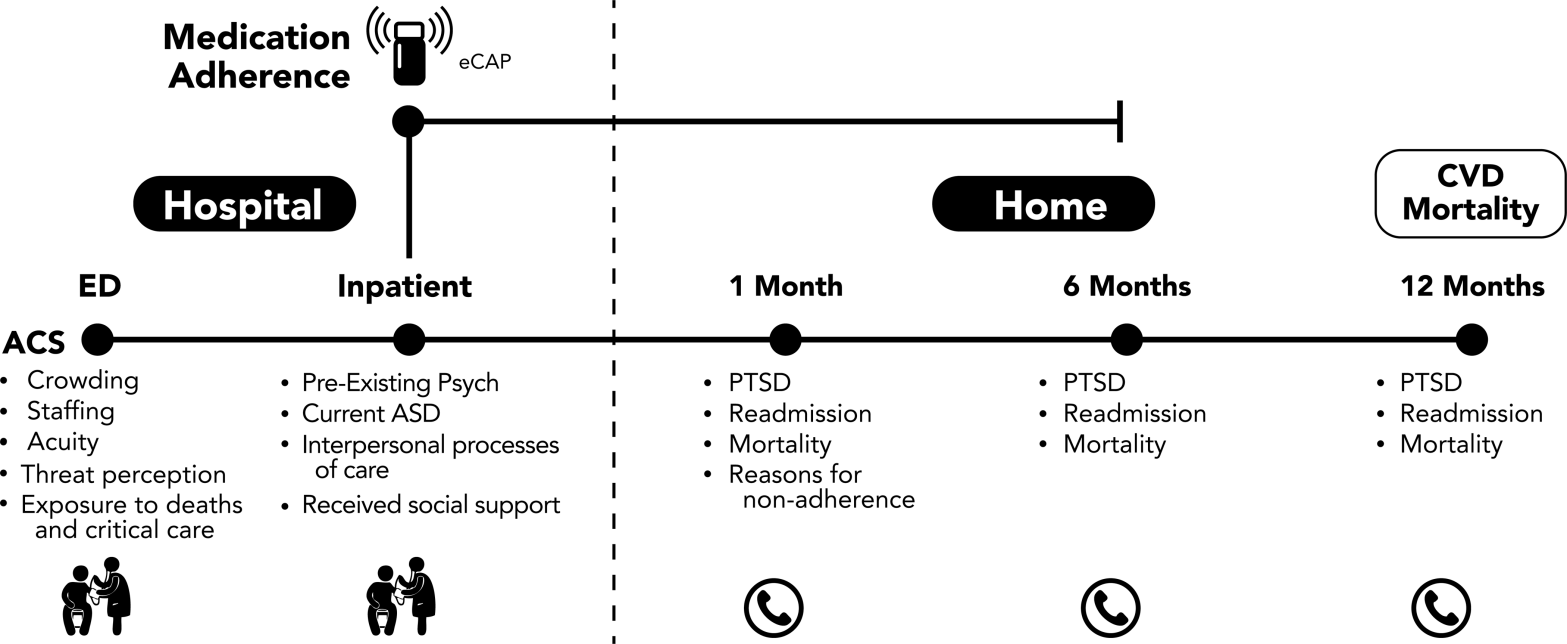
Timetable for ReACH study procedures. The dotted vertical line indicates the transition between study phases occurring in the hospital versus at home. ED = emergency department. CVD = cardiovascular disease. ACS = acute coronary syndrome. ASD = acute stress disorder. PTSD = post-traumatic stress disorder. The eligibility screening and informed consent processes occur at the ED in the left-most time point on the timeline. Baseline assessments occur at the in-hospital ED (T1) and inpatient (T2) time points. Outcome variables are measured from the inpatient time point until up to 6 months for medication adherence and at the 1-month (T3), 6-month (T4), and 12-month (T5) time points for the other study outcome measures.

Potential participants are screened by a team of trained research assistants (RAs) based on the ED E-Track electronic record and physician referrals. After the researchers explain the study and answers any questions that arise, patients who choose to participate provide informed consent. All study protocols have been approved by the Columbia University Medical Center Institutional Review Board. The first phase of the study occurs in the ED (Time 1; T1) and consists of identification of potential participants, informed consent for the main study (and the ancillary, medication adherence study if eligible), and brief interviews about their cardiac event symptoms and current perceptions of personal threat related to the ACS event and the ED itself. Medical record numbers of enrolled participants are recorded so that patients can be tracked as they are transferred to an inpatient bed. The second phase of the study occurs after participants are admitted to inpatient care (Time 2; T2), and consists of the remainder of the baseline interview about psychosocial and interpersonal factors. For participants who are discharged prior to completing the second phase of the baseline questionnaire, the interview is completed by telephone. The third through fifth phases of the study are telephone interviews conducted 1 month (Time 3; T3), 6 months (Time 4; T4), and 12 months (Time 5; T5) after enrollment with participants or their close contacts. The 1-month interview assesses PTSD symptoms using the gold standard screening instrument for PTSD, hospital readmission/mortality (family report), as well as extent of and reasons for cardiovascular medication nonadherence. The 6-month and 12-month telephone interviews are largely identical to the 1-month interview. The ancillary adherence study consists of up to 6 months of electronically-monitored cardiovascular medication adherence beginning at discharge from the hospital; patients are asked to monitor aspirin or if not prescribed then another cardiovascular medication from a bottle with an electronic pillcap. Figure 2 presents the study timetable for the main and ancillary studies.

Study recruitment began in November 2013. The target enrollment of 1,741 participants was achieved in May 2017 with a mean enrollment rate of 2.27 patients per day (*SD* = 1.35). The last of the 1,741 participants completed the 12-month phase of the study in May 2018. Of those 1,741 participants, 730 participants were sent electronic bottle cap (eCAP) devices (see Measures section below) for participation in the ancillary medication adherence study. (This number is necessarily considerably lower than the total number of participants in part because the ancillary study began after the main study.)

### Eligibility criteria

Eligible participants are English-or-Spanish-speaking adults aged 18 years or older who present to the ED with symptoms of a suspected NSTEMI or UA. NSTEMI is defined by a typical rise and gradual fall (troponin levels) or more rapid rise and fall (creatine kinase MB levels) of biochemical markers of infarction with one of the following in the absence of ST elevations: (a) ST-segment depression; (b) T wave abnormalities; (c) ischemic symptoms without ST-segment depression or T wave abnormalities in the presence or absence of chest discomfort (unexplained nausea and vomiting or diaphoresis; persistent shortness of breath; unexplained weakness, dizziness, lightheadedness, or syncope). UA is defined by angina pectoris (or equivalent type of ischemic discomfort) with no biochemical evidence of MI and any of the following within the 6 weeks prior to admission: (a) angina occurring at rest that is prolonged, usually longer than 20 minutes; (b) new-onset angina of least class II severity according to the Canadian Cardiovascular Society criteria; (c) recent worsening of angina reflected by an increase in severity of at least class I to at least class II according to the Canadian Cardiovascular Society criteria.

Patients are excluded from participation if they are unable to complete the baseline assessment (ED and inpatient study phases) within 1 week of hospitalization, do not have reliable phone or email access, are deemed unable to comply with the protocol by self-selection or by indicating during screening an inability to complete all tasks (due to cognitive impairment or current alcohol or substance abuse problems), are deemed to need psychiatric intervention within 72 hours, are prisoners, or are unavailable for follow-up (e.g., life expectancy < 1 year, about to leave United States). Patients are excluded from the ancillary medication adherence study if they are not prescribed aspirin or another cardiovascular medication, are unable to self-administer medications (e.g., live in nursing home), or are concerned that taking a cardiovascular medication from a separate pill bottle will disrupt their medication-taking habits (e.g., patients that prefer to use only pillboxes).

### Measures

#### Phase 1: In the ED (T1)

After consenting patients provide authorization to release medical records, they then provide demographic information and complete an interview-administered questionnaire assessing perceptions of ED crowding and the ACS event, including assessments of current threat perceptions. Participants’ physiological arousal is assessed using each patient’s mean and standard deviation of heart rate, blood pressure, and respiratory rate using ED medical record data. Current threat perceptions in the ED are assessed by participant self-report (e.g., “I am worried that I am dying,” “I feel helpless,” “I think this event will have a big impact on my life”; Ozer, Best, Lipsey, & Weiss, 2003). This measure of ED threat perceptions demonstrates evidence of convergent validity in that it correlates positively with perceptions of relevant ED factors (stress, crowding), and it has shown good internal consistency reliability and stability over time (Cornelius et al., 2018). Finally, intensity and duration of pain are self-reported. Electronic records are used to objectively assess ED variables, and ED crowding metrics such as the Emergency Department Work Index (EDWIN) and National Emergency Department Overcrowding Scale (NEDOCS) are calculated (McCarthy et al., 2008; Weiss et al., 2005). Each patient’s exposure to critical care is recorded, including any ED trauma activations and ED deaths that occur during each participant’s recorded time in the ED.

#### Phase 2: Inpatient (T2)

Characteristics of doctor-patient communication during ED evaluation are assessed during the inpatient interview using The Interpersonal Processes of Care Survey (Stewart, Nápoles-Springer, Gregorich, & Santoyo-Olsson, 2007). Patient-doctor discordances in race, ethnicity, and first language are assessed. Social support in the ED is assessed by participant report of who was present with them in the ED, as well as the degree to which the interpersonal support provided to them was appropriate for the event, whether the patient chose to receive the support, and the quality of the participant’s relationship with the support provider. Due to the study’s specific focus on the consequences of ACS-induced PTSD, previous PTSD not due to NSTEMI/UA is also assessed to avoid potential confounds. This is measured with the PTSD Checklist-Civilian (PCL-C; Weathers, Litz, Herman, Huska, & Keane, 1993). The PCL-C has shown evidence of appropriate convergent and discriminant validity, excellent internal consistency reliability, and adequate test-test reliability (Ruggiero, Ben, Scotti, & Rabalais, 2003). Furthermore, given the unique variance in health outcomes explained by depressive symptoms during hospitalization, depressive symptoms are measured using the Patient Health Questionnaire (PHQ-8; Kroenke et al., 2009) to include as a control variable in primary outcome analyses. This measure has been shown to have adequate validity and internal consistency reliability in previous samples (Kroenke et al., 2009; Pressler et al., 2011). Patients also complete the Acute Stress Disorder Scale (ASDS; Bryant, Moulds, & Guthrie, 2000) to measure early symptoms of distress that may indicate risk for developing PTSD. This measure has been demonstrated to be a sensitive predictor of development of subsequent PTSD and to have excellent internal consistency reliability and strong test-retest reliability (Bryant et al., 2000). Additionally, the following items and scales are collected at T2: recall of ED threat perceptions (parallel to ED threat perception items administered at T1), the Pittsburgh Sleep Quality Index (PSQI; Buysse, Reynolds III, Monk, Berman, & Kupfer, 1989), the Perceived Stress Scale (PSS; Cohen, Kamarck, & Mermelstein, 1994), the International Physical Activity Questionnaire (IPAQ; Craig et al., 2003), and the Life Events Checklist (LEC; Gray, Litz, Hsu, & Lombardo, 2004).

Immediately after discharge, patients enrolled in the ancillary medication adherence study are mailed an electronic bottle cap (eCAP, Information Mediary Corp., Ottawa, Canada) that automatically records openings of medication bottles. For up to 6 months after the initial ACS event, patients are expected to take aspirin or one of their other cardiovascular medications, from a bottle with an eCAP. Participants are contacted to remind them to mail back the eCAP bottle, and for the purposes of this observational study, no counseling about medication adherence is administered.

#### Post-discharge follow-up (T3–T5)

The primary outcomes for this observational study are ACS recurrence and mortality. Proactive medical record searches are conducted daily for all study participants to identify new ED visits and/or hospital readmissions. Suspected ACS events are also identified by participant self-report and then confirmed using hospital records with an adjudication process involving a research nurse and 2 study cardiologists. All-cause mortality is queried by calling alternative contacts, and mortality is confirmed by automatic checking of the social security death index (SSDI) as part of routine management of electronic medical records. Additionally, in-hospital deaths are confirmed via hospitalization records.

The primary study exposure of interest is suspected ACS-induced PTSD. During the 1-month telephone interview, PTSD symptoms related to the suspected ACS event and its treatment are assessed using the PCL-specific for the cardiac event.

The primary mechanism for the hypothesized association of PTSD with ACS recurrence and mortality risk is electronically-measured medication nonadherence. The extent of and reasons for nonadherence are also assessed by self-report. Extent of nonadherence to cardiovascular medications is assessed during the 1-month telephone interview (T3) using a single item from the Coronary Artery Risk Development in Young Adults (CARDIA) study, “In the past month, how often did you take your heart medications as your doctor prescribed?” scored from 1, “less than half of the time (<50%),” to 5, “all of the time (100%)” (Cutter et al., 1991), and by using a 3-item scale psychometrically developed by Voils and colleagues (Voils et al., 2012). Aversive cognitions towards cardiovascular medications are assessed at 1 month (T3) by asking patients: “How often did” 1) “you miss your heart medication because you did not want to be reminded of your heart problem”, 2) “thinking about your heart medication make you feel nervous or anxious”, and 3) “thinking about your heart medication make you think about your risk for future heart problems.” Patients respond using a 5-point Likert scale scored from 1, “never,” to 5, “all of the time.” These items were based directly on the Enduring Somatic Threat theory framework (Edmondson, 2014).

### Statistical approach

To test whether suspected ACS-induced PTSD is associated with increased secondary risk at 1 year, we will use a Cox proportional hazards model with 1-month PTSD assessment (PCL ≥ 33) predicting subsequent acute coronary heart disease event (non-fatal myocardial infarction or hospitalization for unstable angina) or all-cause mortality with adjustment for demographic and clinical variables, as well as depression (PHQ ≥ 10) (Kroenke et al., 2009). The PCL cut point of 33 is used due to its being in the middle of the lower (30) and upper (35) bounds of the range of cut points suggested by the National Center for PTSD for a sample of civilians in a primary care setting or the general population, and was the estimated cutoff associated with increased recurrence risk in the 2013 meta-analysis. If there is a statistically significant association between suspected ACS-induced PTSD and secondary risk, we will determine whether medication nonadherence partially mediates the association between PTSD symptoms and MACE/ACM by running a mediation test with bootstrapping. Specifically, the predictor in this model will be PTSD symptoms assessed at the 1-month interview (T3), the proposed mediator will be electronic adherence data measured prior to the 1-month interview, and the outcome will be occurrence of the secondary risk health events (described above) after the 1-month interview until the end of the study. Regarding the third aim, multiple regression models will be used to test the associations of patient, physician, and ED factors with subsequent 1-month PTSD symptoms induced by suspected ACS. As an exploratory aim, we will determine whether patients who “rule out” for ACS are at similarly increased risk of PTSD and adverse CVD outcomes in a Cox proportional hazards regression that compares survival functions for patients with confirmed ACS versus those who rule out for ACS at baseline.

### Sample size determination

#### Sample for testing links among ED factors, PTSD, risk of mortality and recurrent MACE

We expected that 8% to 10% of participants would have a cardiac event recurrence/ACM event during the year following their index cardiac event. In our previous study, 10.4% had a cardiac event recurrence/ACM in the year following their index cardiac event. The sample size calculations are based on the conservative estimate of 8% cardiac event recurrence/ACM and a 2-tailed test at alpha = 0.05. Although we anticipated higher retention rates, to provide conservative sample size estimates, we allowed for 15% of participants to be unavailable for follow-up at 1 year (although medical record data for the primary outcome will be available for almost all participants). Based on prior data, we anticipated that 10% to 15% of participants would have PTSD at the 1-month follow-up contact. Based on these estimates, we planned to enroll 1,741 participants, which we expect to yield a final sample size of 1,488 or larger. Of the 1,741, we expected that 1,567 would complete the 1-month assessment. For the association between PTSD incidence at 1 month and a new cardiovascular event/ACM in the 1 year after enrollment (hypothesis 1), the detectable hazard ratio is 2.39 assuming that the incidence of PTSD is 15% at 1 month.

### Preliminary results

Table 1 shows the baseline characteristics of patients recruited to date. Participants are racially and ethnically diverse. They demonstrate a range of severity of their ACS conditions, medical comorbidities, prior PTSD symptoms, and depressive symptoms. Overall, the sample is characterized by a wide age range, an expected sex distribution, substantial racial/ethnic diversity, low socioeconomic status, and high medical and psychological comorbidity burden.

## Discussion

Given that hundreds of thousands of patients present for ACS each year, it is imperative to determine the extent to which ACS is associated with the development of PTSD and the extent to which this medical event-induced form of PTSD—in addition to being psychologically debilitating in its own right—is associated prospectively with increased risk of CVD event recurrence and mortality. Then, as a next step, it is essential to examine the mechanisms by which ACS-induced PTSD contributes to secondary risk. One key mechanism may be nonadherence to secondary prevention medications.

The study design has several strengths. First, it benefits from assessing acute stress exposure at the critical time of the ED admission without causing additional, undue burden to patients or altering the course of care or medication habits. Second, it includes a large and diverse longitudinal cohort, which improves generalizability of findings to the larger population of NSTEMI/UA patients across the United States as well as to the even larger number of patients who present to the ED with ACS symptoms but are ruled out, particularly in racially and ethnically diverse, low-SES settings. Third, there is a high quality of measurement for all key variables, including the adjudication of ACS status by expert cardiologists, the objective measure of cardiovascular medication adherence, and attention to potential confounds (i.e., non-ACS-induced PTSD and depression at baseline). Fourth, the study is designed to identify factors that are most important to target for future interventions designed to offset the expected risk of cardiac recurrence, mortality, and PTSD in NSTEMI/UA patients.

The study has some limitations. Evidence of mediation does not definitively prove causality. That is, a causal pathway is not proven if medication nonadherence mediates the association between PTSD symptoms and health outcomes. Nevertheless, the longitudinal design and attention to confounds (e.g., PTSD symptoms unrelated to the ACS event, ACS severity, demographic characteristics) lend credence to a causal interpretation of findings. A second limitation is that the study’s 12-month design precludes the possibility of following these same patients for longer periods after their potentially traumatic experience in the ED. Nevertheless, research suggests that ED factors do influence medical outcomes within 1 year (Pines et al., 2009; Shen & Hsia, 2011). A third limitation is that PTSD is assessed only by self-reported measures and not by psychiatric interview in this study. However, with respect to the gold standard measure for assessing PTSD, the clinician-administered PTSD scale (CAPS; Blake, 1990), the PCL is a PTSD screening tool that has a valid symptom severity index (Ruggiero et al., 2003). Furthermore, if PCL symptoms predict increased cardiac risk, then there may be reason for physicians to use this easily administered measure during the course of clinical care for their ACS patients, irrespective of whether patients meet the diagnostic threshold for PTSD. Finally, it should be noted that the present study focuses on medication adherence, which is just one of several potential mechanisms by which ACS-induced PTSD may influence health outcomes. Other examples of potential mechanisms include autonomic imbalance, low physical activity, and disrupted sleep (for a review, see Edmondson & von Kanel, 2017).

## Conclusion

This prospective observational study will be the most definitive study to date of PTSD as a psychological reaction to an acute cardiac event, and the short-term psychological outcomes and long-term cardiovascular outcomes associated with PTSD, including subsequent MI and death. It will also provide insight into the ED and hospital characteristics that may influence the development of PTSD after acute cardiovascular events. In providing an overview of our study design and methods, we hope to provide future investigators with a model of the assessment of psychological and secondary cardiovascular risk following ACS events in an acute setting. If our hypotheses are supported, we will have identified an important modifiable secondary risk factor (ACS-induced PTSD) that may be preventable through changes to the way patients are evaluated and treated in the ED and hospital. Although many ED factors are largely modifiable, no accepted guidelines exist for managing ED factors that can influence psychological outcomes. Further, these results may shed light on the pernicious problem of nonadherence to secondary-prevention cardiovascular medications, and point to novel psychologically oriented interventions to improve adherence and secondary risk (as well as quality of life) in survivors of acute cardiovascular events.

## Supplementary Material

Analysis Package

Replication Package

## Figures and Tables

**Table 1:** Demographic and clinical participant characteristics.

**Demographic information**	

Age, mean in years (SD)	60.54 (13.28)
Female	818 (47.0%)
Black	397 (24.0%)
Hispanic	1,003 (59.4%)
Education (completed high school)	1,137 (65.4%)
Current partner	751 (43.5%)
Current health insurance	1,537 (89.5%)

**Medical information**	

ACS confirmed: NSTEMI	311 (17.9%)
ACS confirmed: UA	230 (13.2%)
Prior cardiac event*	537 (30.8%)
Atherosclerotic disease	199 (11.7%)
GRACE score, mean (SD)	93.80 (29.98)
Charlson comorbidity index, mean (SD)	1.78 (1.98)

**Psychiatric conditions at baseline**	

Non-ACS-induced PTSD symptoms, mean (SD)	26.81 (13.71)
Baseline depressive symptoms, mean (SD)	6.61 (5.90)

* Record of prior confirmed myocardial infarction, coronary artery bypass grafting (CABG), or percutaneous coronary intervention (PCI).
