# Supplementary material for: The Impact of Cardiac-induced Post-traumatic Stress Disorder Symptoms on Cardiovascular Outcomes: Design and Rationale of the Prospective Observational Reactions to Acute Care and Hospitalizations (ReACH) Study
Source: Health Psychol Bull. Author manuscript; Available in PMC 2020 Jan 3. (PMC6941797; doi:10.5334/hpb.16)
Supplement: Analysis Package [file NIHMS1062792-supplement-Analysis_Package.docx]

**Analysis Package**

Below we provide the SPSS syntax used to test the primary aims of the study. Because data collection for this project is still ongoing and because the associated manuscript fully covers the design and rationale of the project (rather than results of data analysis), complete raw data files are not yet available.

**Guide to variable names**

- **Age:** Age in years (continuous variable)
- **Charlson:** Charlson comorbidity index (continuous variable)
- **CHT_D07_ACSConfirmed:** The patient experienced a confirmed acute coronary syndrome (ACS) event (dichotomous variable: 1 = yes, 0 = no)
- **Edwin:** The Emergency Department Work Index (EDWIN), a measure of emergency department crowding (continuous variable)
- **Gender:** Gender (1 = male, 2 = female, 3 = other) (categorical variable)
- **Grace:** The Global Registry of Acute Coronary Events (GRACE) risk score (continuous variable)
- **MACE_or_ACM_365d_bin:** Major acute coronary event (MACE) or all-cause mortality(ACM) at one-year after the acute coronary syndrome (ACS) event (dichotomous variable: 1 = presence, 0 = absence)
- **MACE_or_ACM_365d_daysto:** Number of days to incidence of MACE or ACM at one-year after the ACS event (continuous variable)
- **Days_to_MACE_or_ACM:** Number of days between the 1-month interview and either

a) an event (when MACE_or_ACM_365d_bin=1) or b) the date of last contact when person was known not to have had an event (when MACE_or_ACM_365d_bin=0) (continuous variable)

- **MedAdhere:** Proportion of days per month prior to the 1-month interview that participants took the correct number of aspirin pills (continuous variable)
- **PCL_Combo_im_01m:** PTSD score 1 month after emergency department visit for suspected ACS; a combination of PCL-C and PCL-5 scales using imputed missing values (continuous variable)
- **PCL_Combo_im_posscreen:** Grouping variable of PTSD score (combination of PCL-C and PCL-5 scales) using imputed missing values. Scores are evaluated with respect to whether or not they are greater than or equal to the cut point of 33 (dichotomous variable: 1 = yes, 0 = no)
- **PHQ_ScoreGE10_im:** Grouping variable of PHQ score using imputed missing values. Scores are evaluated with respect to whether or not they are greater than or equal to the cut point of 10 (dichotomous variable: 1 = yes, 0 = no)
- **Race:** American Indian/Native American, Asian, Hawaiian/Pacific Islander, Black, White, Other race, Two or more races (categorical variable)
- **Ethnicity:** Hispanic or Not Hispanic (categorical variable)

**Aim 1: Association of post-traumatic stress disorder (PTSD) induced by suspected acute coronary syndrome (ACS) with major acute coronary event (MACE) or all-cause mortality (ACM)**

**[Syntax below to be run in SPSS software]**

COXREG MACE_or_ACM_365d_daysto

/STATUS=MACE_or_ACM_365d_bin(1)

/METHOD=ENTER Age Gender Grace Charlson CHT_D07_ACSConfirmed

/METHOD=ENTER PHQ_ScoreGE10_im PCL_Combo_im_posscreen

/PRINT=CI(95)

/CRITERIA=PIN(.05) POUT(.10) ITERATE(20).

**Aim 2: Mediation to test whether medication nonadherence mediates any association of suspected ACS-induced PTSD with MACE or ACM within 12-months after discharge from the emergency department**

**[Syntax below to be run in SAS software. The proposed predictor uses adherence data collected prior to the 1-month assessment of PTSD. The CV/ACM events are measured starting after the 1-month PTSD assessment. Thus, the test of mediation is a prospective analysis.]**

options ls=**116** ps=**79** nofmterr noxwait formchar='7C2DDAC2BFC32BB4C0C1D9'x;*DOS;

title "Bootstrap_Mediation: Testing mediation of medication adherence in association between PTSD symptoms and MACE/ACM";

**data** analysis_dataset; set analysis_dataset;

keep PCL_Combo_im_01m MACE_or_ACM_365d_bin

MedAdhere

race ethnicity gender

grace charlson PHQ_ScoreGE10_im CHT_D07_ACSConfirmed;

**run**;

**data** analysis_dataset; set analysis_dataset;

if PCL_Combo_im_01m ne **.** ;

if MACE_or_ACM_365d_bin ne **.** ;

if MedAdhere ne **.** ;

if race ne **.** ;

if race ne '.M' ;

if ethnicity ne **.** ;

if ethnicity ne '.M' ;

if gender ne **.** ;

if age ne **.** ;

if grace ne **.** ;

if charlson ne **.** ;

if PHQ_ScoreGE10_im ne **.** ;

if CHT_D07_ACSConfirmed ne **.** ;

**run**;

*** The bootstrap test of mediation begins here;

%let Analysis_Dataset= analysis_dataset;

%let N_Bootstrap_Samples=1001; * The number of bootstrap samples can be modified here;

%let Starting_Random_Number=631498735; * This can be modified (must be integer <2147483648);

**proc** **means** data= analysis_dataset;

var PCL_Combo_im_01m MedAdhere MACE_or_ACM_365d_bin

race ethnicity gender

age grace charlson PHQ_ScoreGE10_im CHT_D07_ACSConfirmed;

output out=N_obs N=N_obs;

**run**;

**proc** **print**;

**data** _null_; set N_obs;

call symput("N_obs",compress(put(N_obs,**7.**)));

**run**;

**data** temp1; set analysis_dataset;

_Record_=_n_;

**run**;

**data** temp2(drop=i);

do Bootstrap_Sample=**1** to &N_Bootstrap_Samples;

do i=**1** to &N_obs;

_Record_=max(**1**,ceil(&N_obs * ranuni(&Starting_Random_Number)));

output; end; end;

**run**;

**proc** **means**;

**run**;

**proc** **sort**; by _record_;

**run**;

**data** BootStrapDatabase; merge temp2 temp1; by _Record_;

**run**;

**proc** **sort**; by Bootstrap_Sample _Record_;

**run**;

*** We now have the specified number of bootstrap samples;

*** The next step is to run the analysis on each bootstrap sample;

*** General linear model for A path: X predicting M, which is a continuous variable;

**proc** **glm** data=BootStrapDatabase; by Bootstrap_Sample;

class race ethnicity gender;

model MedAdhere = PCL_Combo_im_01m

race ethnicity gender age grace charlson PHQ_ScoreGE10_im CHT_D07_ACSConfirmed

/ solution clparm;

ods output ParameterEstimates=ParameterEstimates_Eq_1;

**run**;

*** Regression for B path: X and M predicting Y, which is a dichotomous variable;

**proc** **phreg** data=BootStrapDatabase;  by Bootstrap_Sample;

class race ethnicity gender;

model Days_to_MACE_or_ACM*MACE_or_ACM_365d_bin(0) = PCL_Combo_im_01m MedAdhere

              race ethnicity gender age grace charlson PHQ_ScoreGE10_im CHT_D07_ACSConfirmed;

ods output ParameterEstimates=ParameterEstimates_Eq_2;

**run**;

**data** a_path;  set ParameterEstimates_Eq_1;

  if Parameter='PCL_Combo_im_01m' then do;

     a_path=Estimate;  se_a_path=StdErr;  output;  end;

  keep Bootstrap_Sample a_path se_a_path;

**run**;

**data** b_path; set ParameterEstimates_Eq_2; by Bootstrap_Sample;

retain b_path se_b_path c_prime_path se_c_prime_path;

if Parameter='PCL_Combo_im_01m' then do;

c_prime_path=Estimate; se_c_prime_path=StdErr; end;

if Parameter='MedAdhere' then do;

b_path=Estimate; se_b_path=StdErr; end;

if last.Bootstrap_Sample then output;

keep Bootstrap_Sample b_path se_b_path c_prime_path se_c_prime_path;

**run**;

*** Compute signs of A path and B path for each bootstrap sample;

**data** final; merge a_path b_path; by Bootstrap_Sample;

sign_a_path=sign(a_path); sign_b_path=sign(b_path);

**run**;

**proc** **means**;

**run**;

*** Test for significance of the mediating effect (a path * b path) of the predictor on the outcome by way of the proposed mediator;

**proc** **freq**;

table sign_a_path*sign_b_path;

title2 'The overall percentage for the (-1 a path,-1 b path) cell equals "1 - p-value" for mediation hypothesis';

title3 'assuming that the original a-path and b-path were both negative, as predicted for the current hypothesis (i.e., HIGHER PTSD symptoms predict LOWER medication adherence, and HIGHER medication adherence predicts lower likelihood of MACE/ACM';

**run**;

**Aim 3: Association of patient, physician, and ED factors and 1-month PTSD symptoms induced by suspected ACS**

**[Syntax below to be run in SPSS software. This syntax is one instance pertaining specifically to crowding of the ED using the Edwin crowding score (see “Edwin” underlined below). For all tests of predictors related to the patient, physician, and ED described in the design paper, this syntax can be used by replacing the underlined word with the relevant factor in question.]**

REGRESSION

/DESCRIPTIVES MEAN STDDEV CORR SIG N

/MISSING LISTWISE

/STATISTICS COEFF OUTS R ANOVA COLLIN TOL CHANGE

/CRITERIA=PIN(.05) POUT(.10)

/NOORIGIN

/DEPENDENT PCL_Combo_im_01m

/METHOD=ENTER Edwin Age Gender Race Ethnicity Grace Charlson PHQ_ScoreGE10_im.

**Exploratory Aim: Association of PTSD with MACE/ACM within 12-months after discharge from the emergency department by ACS status (confirmed vs. rule-out)**

**[Syntax below to be run in SPSS software]**

COXREG MACE_or_ACM_365d_daysto

/STATUS=MACE_or_ACM_365d_bin(1)

/STRATA=CHT_D07_ACSConfirmed

/METHOD=ENTER Age Gender Grace Charlson

/METHOD=ENTER PHQ_ScoreGE10_im PCL_Combo_im_GE34

/PRINT=CI(95)

/CRITERIA=PIN(.05) POUT(.10) ITERATE(20).
