# Supplementary material for: The Impact of Cardiac-induced Post-traumatic Stress Disorder Symptoms on Cardiovascular Outcomes: Design and Rationale of the Prospective Observational Reactions to Acute Care and Hospitalizations (ReACH) Study
Source: Health Psychol Bull. Author manuscript; Available in PMC 2020 Jan 3. (PMC6941797; doi:10.5334/hpb.16)
Supplement: Replication Package [file NIHMS1062792-supplement-Replication_Package.docx]

**Replication Package**

**Study consent forms, information forms, and protocols**

Attached are the following study protocol forms with versions in all cases in both English and in Spanish: 1) consent form for the Reactions to Acute Care and Hospitalizations (ReACH) study, 2) HIPAA authorization form, 3) adherence monitoring information sheet, and 4) adherence monitoring instructions for participants.

**Questionnaires**

Attached to this replication package are questionnaires administered to study participants at baseline, 3 months, 6 months and 12 months. The majority of these questionnaires within these packets are publicly available.

The following information is collected at baseline. First, an Enrollment Data Form is collected in Filemaker. Then, baseline questionnaires are administered verbally to participants that include the following (administered in the order listed): 1) Medical Record Release, 2) Participant Contact, 3) Demographics, 4) ACS Symptoms, 5) Emergency Room Perceptions-PRESENT.

The following scales are collected after the ED phase: 1) Emergency Room Perceptions-POST, 2) Acute Stress Disorder Scale (ASDS), 3) Pittsburgh Sleep Quality Index (PSQI), 4) Perceived Stress Scale (PSS), 5) Patient Health Questionnaire (PHQ-8), 6) International Physical Activity Questionnaire (IPAQ), 7) Life Events Checklist (LEC), 8) PTSD Checklist for Diagnostic and Statistical Manual of Mental Disorders (PCL-5).

**Ethical approval forms**

The initial approval letter from the Institutional Review Board (IRB) is attached to this submission.

**Study aims**

As described in the manuscript, the study aims are as follows: (1) to test whether ACS-induced PTSD is associated with increased risk of mortality and recurrent major adverse cardiac events (MACE) at 1 year; (2) to determine whether electronically-measured medication nonadherence mediates any association of ACS-induced PTSD with CVD risk; (3) to identify predictors of ACS-induced PTSD that are related to the patient, the physicians, and the ED environment; (4) and to determine whether patients who “rule out” for ACS are similarly at increased risk of PTSD and adverse CVD outcomes.

**Analysis plans**

Also as described in the manuscript, to test whether suspected ACS-induced PTSD is associated with increased secondary risk at 1 year, we will use a Cox proportional hazards model with 1-month PTSD assessment (PCL ≥ 33) predicting subsequent acute coronary heart disease event (non-fatal myocardial infarction or hospitalization for unstable angina) or all-cause mortality with adjustment for demographic and clinical variables, as well as depression (PHQ ≥ 10). The PCL cut point of 33 is used due to its being in the middle of the lower (30) and upper (35) bounds of the range of cut points suggested by the National Center for PTSD for a sample of civilians in a primary care setting or the general population, and was the estimated cutoff associated with increased recurrence risk in the 2013 meta-analysis. If there is a statistically significant association between suspected ACS-induced PTSD and secondary risk, we will determine whether medication nonadherence partially mediates the association between PTSD symptoms and MACE/ACM by running a mediation test with bootstrapping. Specifically, the predictor in this model will be PTSD symptoms assessed at the 1-month interview, the proposed mediator will be electronic adherence data measured prior to the 1-month interview, and the outcome will be occurrence of the secondary risk health events (described above) after the 1-month interview until the end of the study. As an exploratory aim, we will determine whether patients who “rule out” for ACS are at similarly increased risk of PTSD and adverse CVD outcomes in a sensitivity analysis limited to only those who rule out for ACS at baseline.

**Rationale for determination of sample size with power analysis**

We expected that 8% to 10% of participants would have a cardiac event recurrence/all-cause mortality (ACM) event during the year following their index cardiac event. In our previous study, 10.4% had a cardiac event recurrence/ACM in the year following their index cardiac event. The sample size calculations are based on the conservative estimate of 8% cardiac event recurrence/ACM and a 2-tailed test at alpha=0.05. Although we anticipated higher retention rates, to provide conservative sample size estimates, we allowed for 15% of participants to be unavailable for follow-up at 1 year (although medical record data for the primary outcome will be available for almost all participants). Based on prior data, we anticipated that 10% to 15% of participants would have PTSD at the 1-month follow-up contact. Based on these estimates, we planned to enroll 1,741 participants, which we expect to yield a final sample size of 1,488 or larger. Of the 1,741, we expected that 1,567 would complete the 1-month assessment. For the association between PTSD incidence at 1 month and a new cardiovascular event/ACM in the 1 year after enrollment (hypothesis 1), the detectable hazard ratio is 2.39 assuming that the incidence of PTSD is 15% at 1 month.
